# Supplementary material for: Biochemical-free enrichment or depletion of RNA classes in real-time during direct RNA sequencing with RISER
Source: Nat Commun. 2024 May 24;15:4422. doi: 10.1038/s41467-024-48673-8 (PMC11126589; doi:10.1038/s41467-024-48673-8)
Supplement: Supplementary file 1 — Supplementary Information [file 41467_2024_48673_MOESM1_ESM.pdf]

Supplementary Material

Biochemical-free enrichment or depletion of RNA classes in real-time during direct RNA sequencing with RISER

Alexandra Sneddon[1,2,3], Agin Ravindran[1,2,3], Somasundhari Shanmuganandam[4,5], Madhu Kanchi[3], Nadine Hein[6], Simon Jiang[4,5,7], Nikolay Shirokikh[3]*, Eduardo Eyras[1,2,3]*

[1] EMBL Australia Partner Laboratory Network at the Australian National University, Canberra, ACT 2601, Australia.

[2] Centre for Computational Biomedical Sciences, The John Curtin School of Medical Research, Australian National University, Canberra, ACT 2601, Australia.

[3] The Shine-Dalgarno Centre for RNA Innovation, The John Curtin School of Medical Research, Australian National University, Canberra, ACT 2601, Australia.

[4] Department of Immunity, Inflammation and Infection, The John Curtin School of Medical Research, Australian National University, Canberra, ACT 2601, Australia.

[5] Centre for Personalised Immunology, NHMRC Centre for Research Excellence, Australian National University, Canberra, ACT 2601, Australia.

[6] ACRF Department of Cancer Biology and Therapeutics, The John Curtin School of Medical Research, Australian National University, Canberra, ACT 2601, Australia.

[7] Department of Renal Medicine, The Canberra Hospital, Canberra, ACT 2605, Australia.

* Correspondence to: eduardo.eyras@anu.edu.au, nikolay.shirokikh@anu.edu.au

Supplementary Information

Supplementary Figures

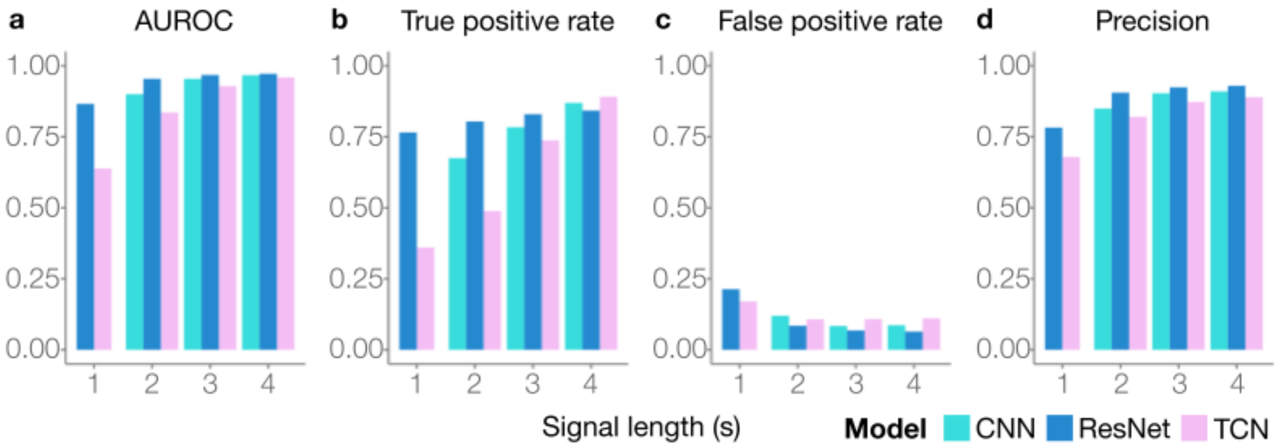

Supplementary Figure 1: Evaluation of candidate RISER models. **a-d,** Model performance on the test set for each candidate input signal length (x-axes) in seconds, color-coded by three convolutional network architectures: vanilla convolutional neural network (CNN) (cyan), residual network (ResNet) (dark blue) and temporal convolutional network (TCN) (pink). We show the area under the receiver operating characteristic curve (AUROC) (**a**), true positive rate (**b**), false positive rate (**c**) and precision (**d**). Source data are provided as a Source Data file.

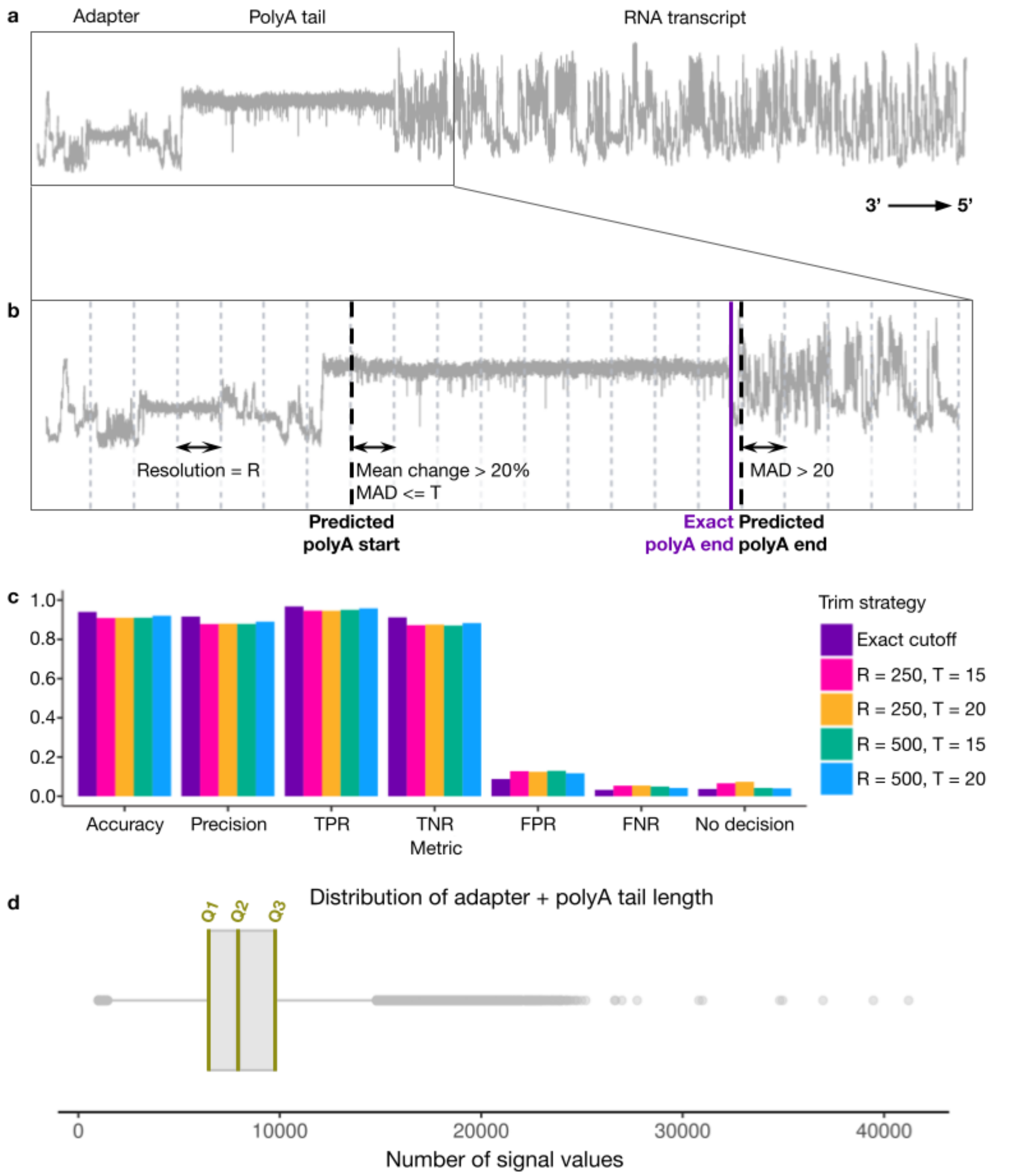

Supplementary Figure 2: Removal of the sequencing adapter and poly(A) tail from the nanopore signal. a, Example DRS signal from the 3' to 5' direction with adapter, poly(A) tail and RNA transcript portions of the signal annotated. **b,** 3' end of the signal shown in (a) with the predicted (black) and actual (purple) boundary between the poly(A) tail and RNA transcript shown, as well as the signal characteristics used for predicting the poly(A) tail position. **c,** Model performance on the mRNA model development test set to classify mRNA or non-mRNA when signals were trimmed at the exact boundary between the poly(A) tail and RNA transcript (purple), as well as by each candidate trimming strategy configuration: resolution (R) = 250 and initial median absolute deviation threshold (T) = 15 (pink), R = 250 and T = 20 (yellow), R = 500 and T = 15 (green) and R = 500 and T = 20 (blue). We show the metrics of accuracy, precision, true (TPR) and false

(FPR) positive rate, true (TNR) and false (FNR) negative rate and the proportion of signals for which a confident prediction could not be made. **d,** Distribution of the number of signal values (x-axis) comprising the adapter and poly(A) tail for the model development dataset. In the box plot, the left and right boundaries of the box are the first and third quartiles, with the median annotated with a line inside the box. The whiskers extend to the maximum and minimum values within 1.5 times the interquartile range. The first and third quartiles, as well as the median, are highlighted green. Source data for c,d are provided as a Source Data file.

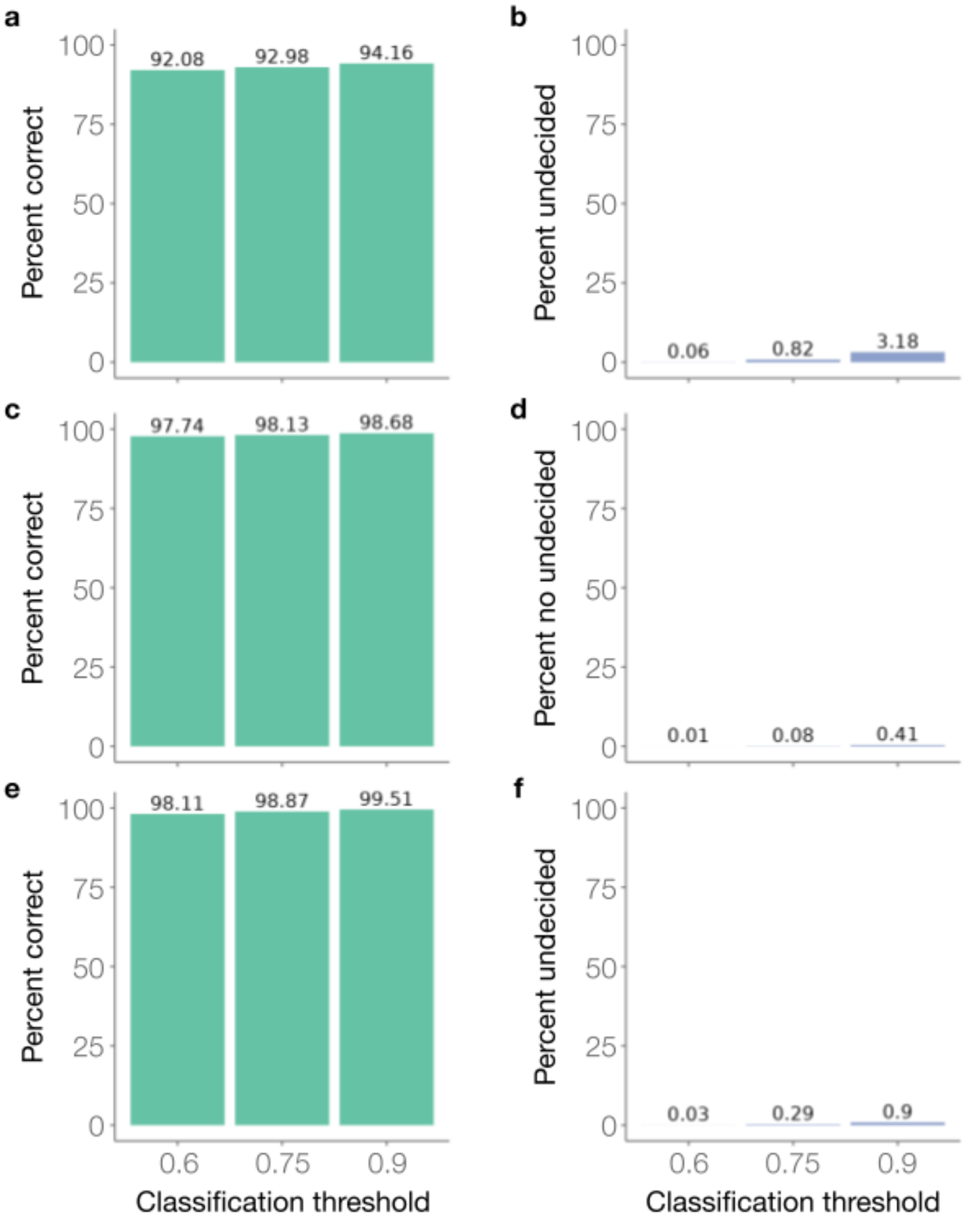

Supplementary Figure 3: Classification threshold optimization. For each candidate classification threshold (0.6: low, 0.75: medium and 0.9: high), we show the percentage of reads that RISER predicted correctly (**a**,**c**,**e**) or was unable to make a prediction for (**b**,**d**,**f**) in the respective test sets for the three RISER models implemented (mRNA: a-b, mtRNA: c-d, globin mRNA: e-f).

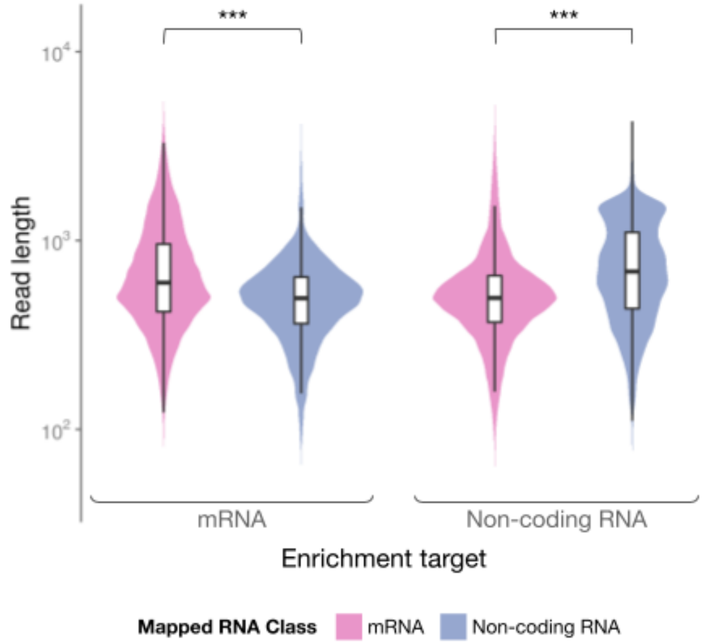

Supplementary Figure 4: RISER reduces the length of off-target reads when used to enrich the target RNA class in a simulated live-sequencing environment. The distribution of read lengths for the mapped RNA class when the RISER target class for enrichment was mRNA (left panel) and non-coding RNA (right panel) in a simulated live-sequencing run of an REH cancer cell line run using MinKNOW playback. The mapped RNA class is color-coded (pink: mRNA, blue: non-coding RNA). For both targets, the read lengths of the mapped RNA classes were compared using a Wilcoxon rank sum test with continuity correction (H_1: on-target > off-target). The probability of superiority (PS) is also shown. PS is the probability that a randomly sampled read from the control condition is longer than a randomly sampled read from the deplete condition (PS close to 0.5 means the lengths are likely to be the same, whereas PS close to 1 means that control lengths are highly likely to be larger) (mRNA target: p-value (p) < 2.2E-308, U1 = 1006637288.0, PS = 0.63, n = 142,677, non-coding RNA target: p < 2.2E-308, U1 = 931154993.0, PS = 0.65, n = 164,489). Outliers were not included. Source data are provided as a Source Data file.

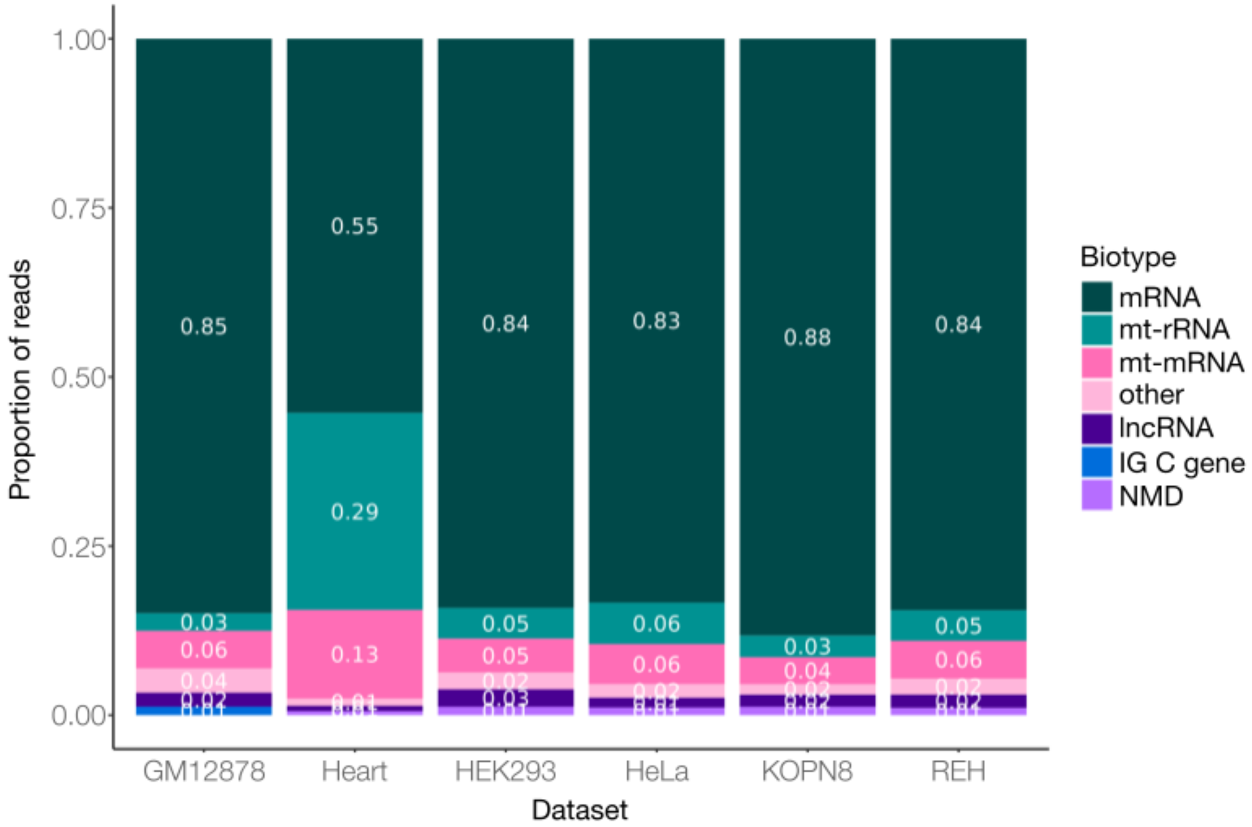

Supplementary Figure 5: Proportion of reads per biotype in standard DRS runs across different cell lines. The top 5 biotypes according to read proportion are displayed for each run, with the remaining biotypes aggregated under “other”. Source data are provided as a Source Data file.

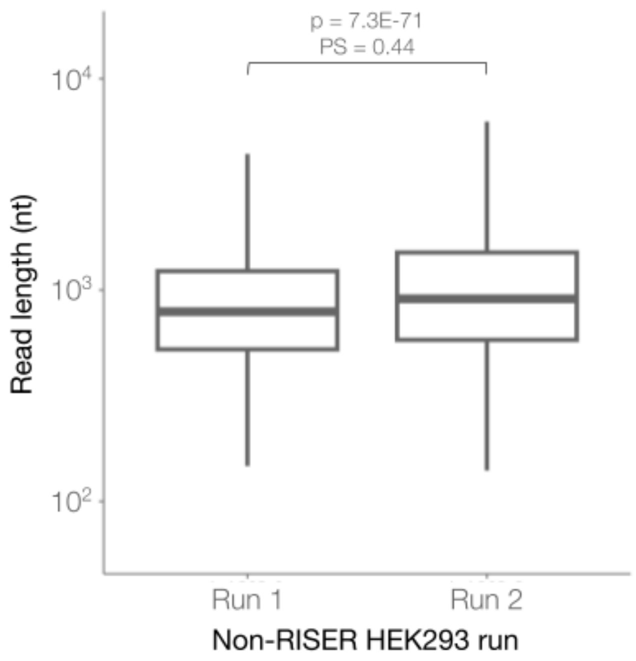

Supplementary Figure 6: Read length distributions in non-RISER sequencing runs of HEK293 cells. Distribution of read lengths (y-axis, log10-scale) for two MinION Mk1B sequencing runs performed without RISER. In the box plot, the upper and lower boundaries of the box are the first and third quartiles, with the median annotated with a line inside the box. The whiskers extend to the maximum and minimum values within 1.5 times the interquartile range. Outliers were not included. The read lengths of each run were compared using a Wilcoxon rank sum test (H1: run1 ≠ run2) (p-value = 7.3E-71, U1 = 73784040.0, *n* = 26,093). The probability of superiority (PS = 0.44) is also shown. PS is the probability that a randomly sampled read from run 1 is longer than a randomly sampled read from run 2 (i.e., PS close to 0.5 means the lengths are likely to be the same, whereas PS close to 0 means that the run 1 lengths are highly likely to be shorter). Source data are provided as a Source Data file.

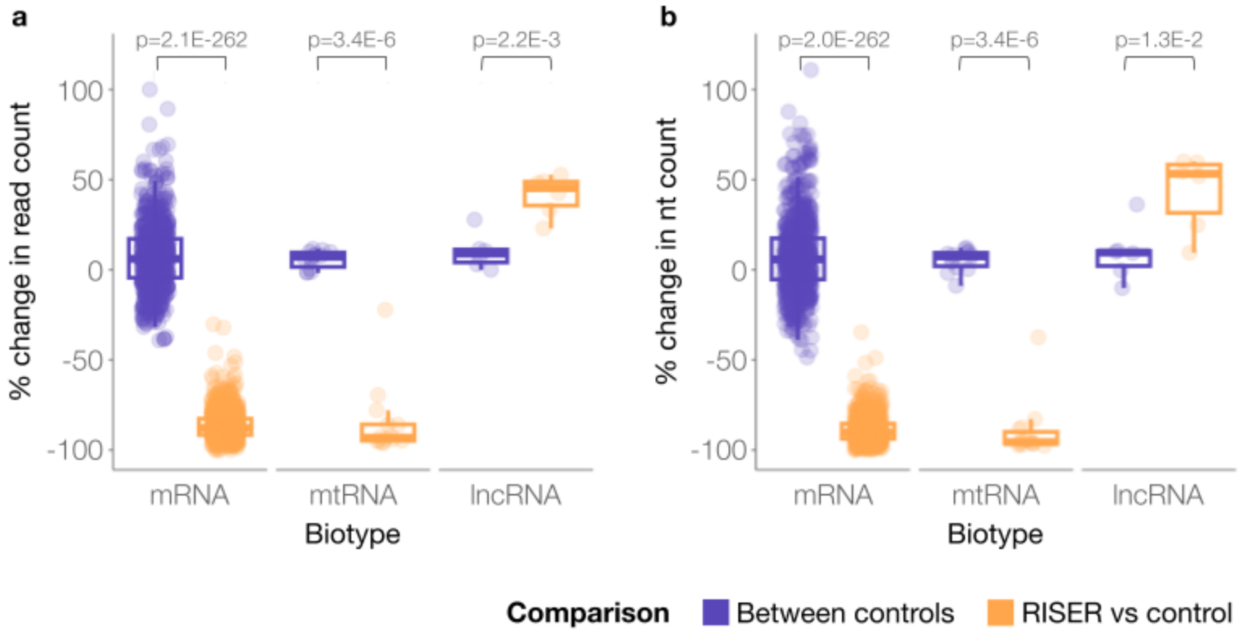

Supplementary Figure 7: Effect of RISER on read and nucleotide counts. Distribution of the percent change in read (**a**) and nucleotide (nt) (**b**) counts (y-axis) with respect to a control run, when RISER was used to deplete mRNA and mtRNA (orange) and for a separate control run (purple). In the box plots, the upper and lower boundaries of the box are the first and third quartiles, with the median annotated with a line inside the box. The whiskers extend to the maximum and minimum values within 1.5 times the interquartile range. For the set of transcripts in each biotype, the percent change in nt or reads using RISER was compared to the (no-RISER) control using a paired Wilcoxon signed rank test (H1 for mRNA and mtRNA: RISER<between controls, H1 for lncRNA: RISER>between controls) (test parameters and statistics in **Supp. Table 8**). For this comparison, lncRNAs that did not overlap with coding exons from protein-coding transcripts were used. Source data are provided as a Source Data file.

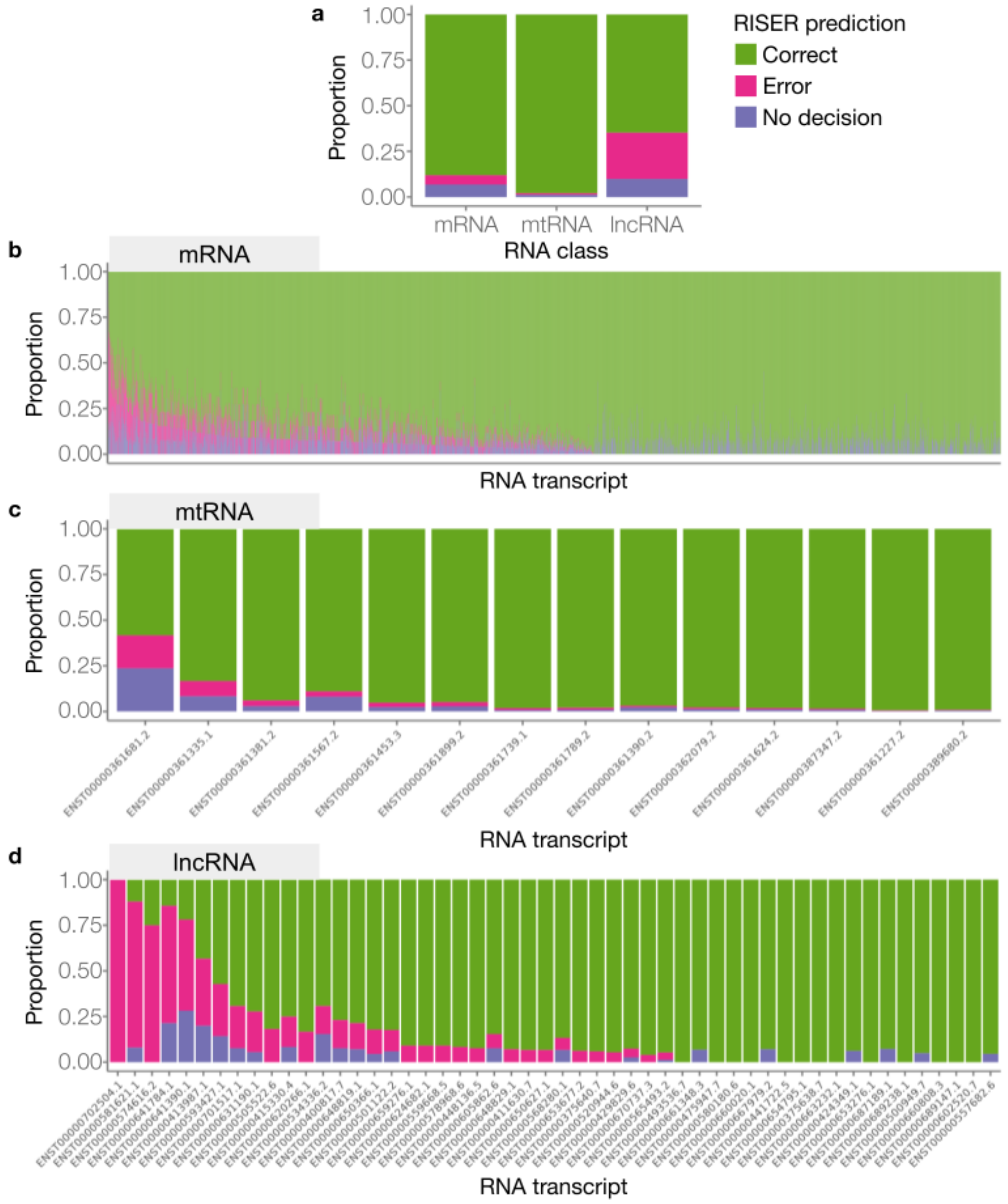

Supplementary Figure 8. mRNA and mtRNA depletion transcript analysis. We represent graphically the proportion of reads (y-axes) correctly classified (green), wrongly targeted by RISER (pink), or for which no decision was made (purple) during the live runs targeting mRNA and mtRNA for depletion. We represent these proportions for all mRNA, mtRNA and lncRNAs (**a**), and for all the individual transcripts from each class: mRNA (**b**), mtRNA (**c**) and lncRNA (**d**). Source data are provided as a Source Data file.

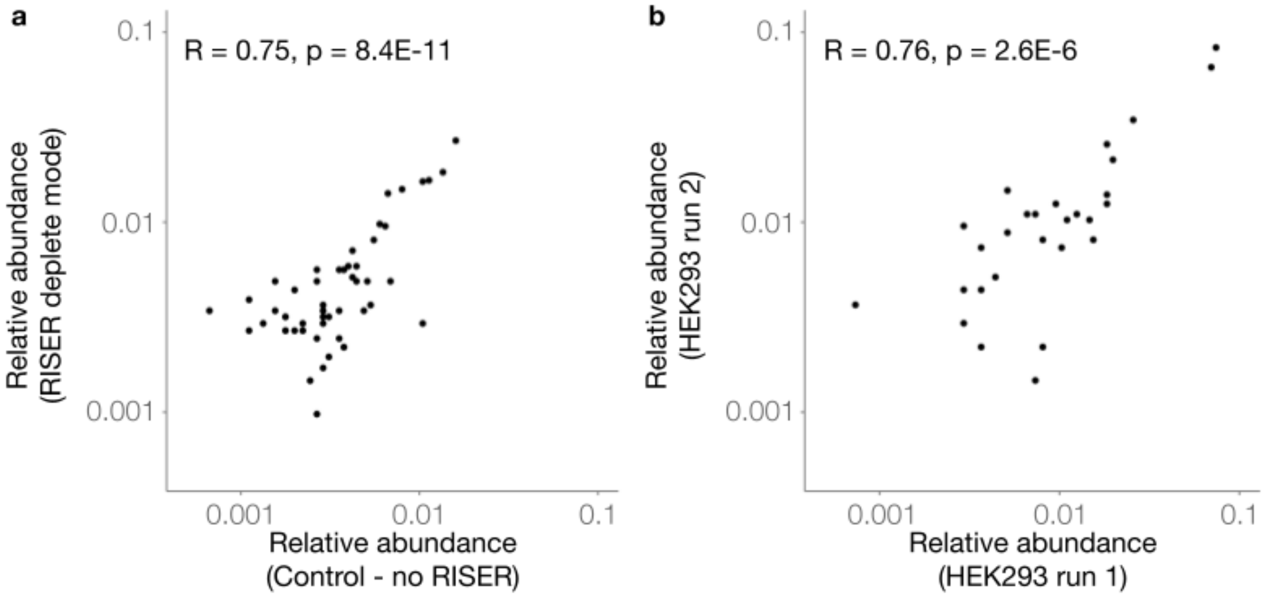

Supplementary Figure 9: Conserved relative abundance of lncRNA after mRNA and mtRNA depletion. a, For each lncRNA (*n* = 53) detected in the control (x-axis) and the RISER deplete mRNA and mtRNA (y-axis) conditions (in the same flow cell sequencing HEK293), we represent their abundance in log10 scale, measured as the proportion of reads mapped to that lncRNA over all reads mapped to all detected lncRNAs. Only lncRNAs with at least 10 reads in either the control or RISER condition were included. The Pearson correlation *(R)* is also shown. **b,** The abundance (calculated as in (a)) of lncRNAs (*n* = 28) measured in two separate sequencing experiments, without RISER, for the same cell line as in (a) (HEK293), considering the same number of reads as in (a), randomly sampled from the same set of transcripts as in (a). The Pearson correlation *(R)* is also shown. HEK293 DRS samples were prepared and sequenced with a MinION Mk1B as described in Methods. Source data are provided as a Source Data file.

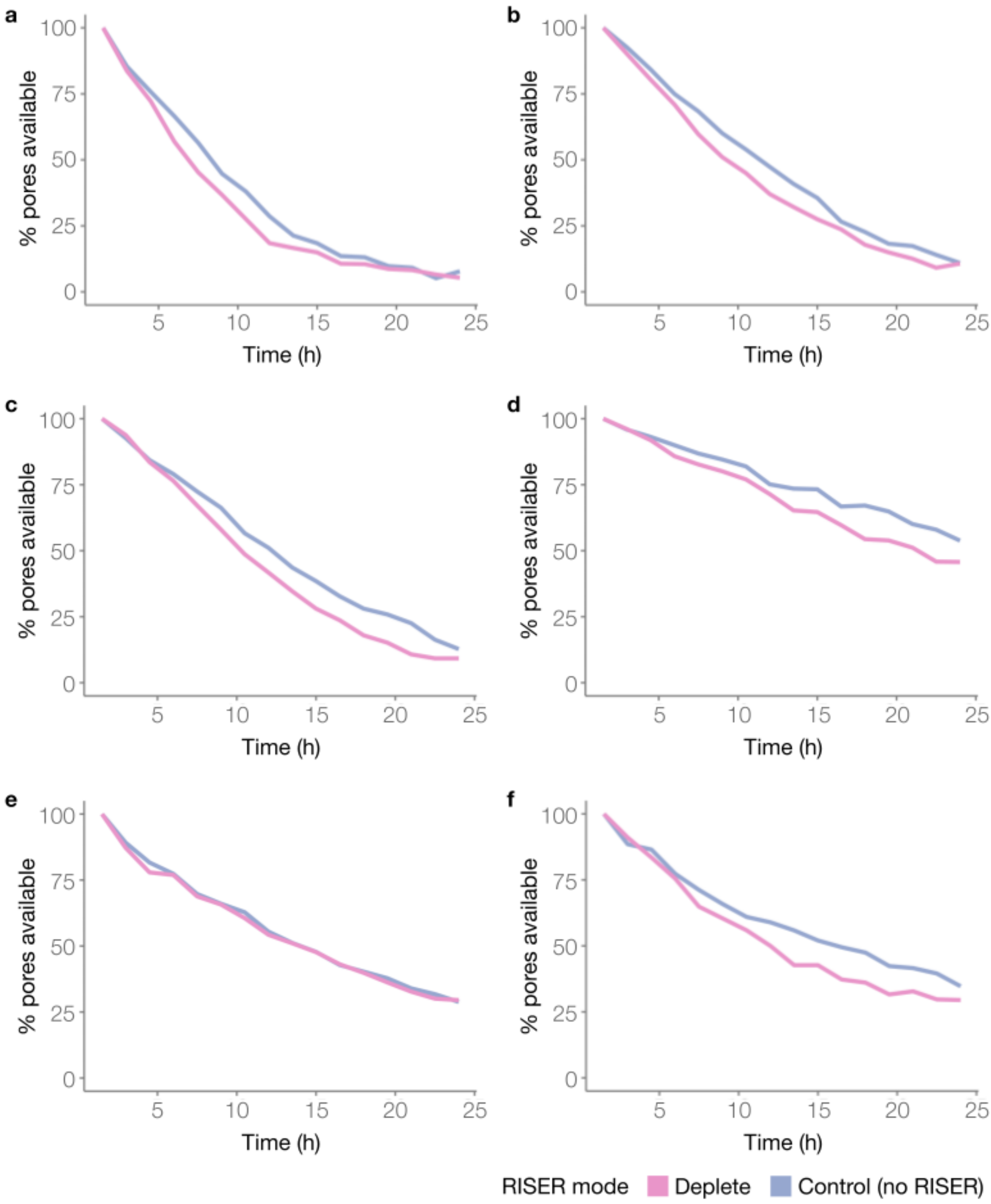

Supplementary Figure 10: Percentage of available pores across 24h DRS runs. We show the pores available over time as a percentage of the initial number of available pores for both RISER (pink) and control (blue) conditions, with RISER depleting mRNA and mtRNA in HEK293 (**a**, **b**) and HeLa (**c**) cell lines, and with RISER depleting globin mRNA in whole blood (**d-f**) samples. HEK293 DRS samples were prepared and sequenced as described in Methods. The HeLa sample was prepared as described in [1] and sequenced as described for HEK293 in Methods. The whole blood samples (**d-f**) were prepared and sequenced as described in Methods, except in (**f**), where total RNA from whole blood was extracted using the PAXgene blood RNA kit (Qiagen) as per manufacturer's instructions. Source data are provided as a Source Data file.

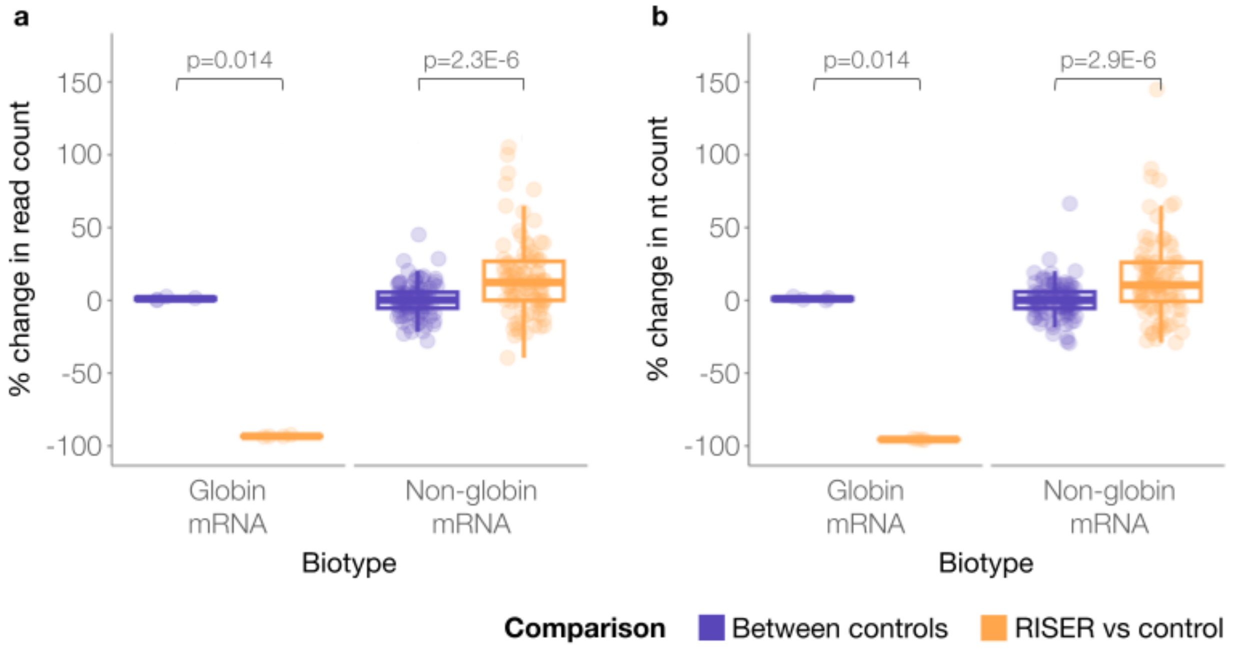

Supplementary Figure 11: Effect of RISER on read and nucleotide counts. Distribution of the percent change in read (**a**) and nucleotide (nt) (**b**) counts (y-axis), relative to a control run, when RISER was used to deplete globin mRNA (orange) and for a separate control run (purple). In the box plots, the upper and lower boundaries of the box are the first and third quartiles, with the median annotated with a line inside the box. The whiskers extend to the maximum and minimum values within 1.5 times the interquartile range. For the set of transcripts in each biotype, the percent change in nt or reads using RISER was compared to the (no-RISER) control using a paired Wilcoxon signed rank test (H1 for globin mRNA : RISER<between controls, H1 for non-globin mRNA: RISER>between controls) (test parameters and statistics in **Supp. Table 8**). Source data are provided as a Source Data file.

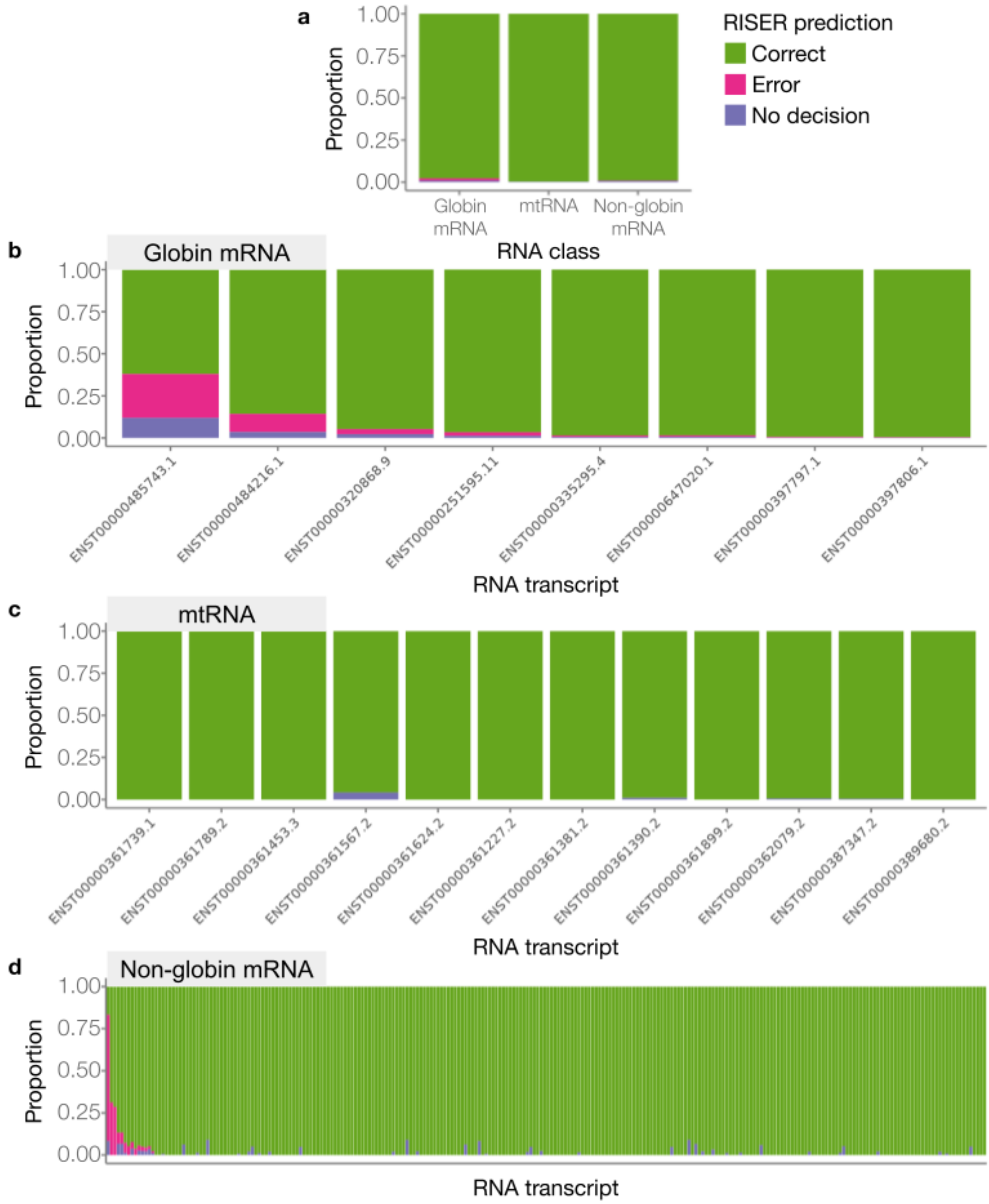

Supplementary Figure 12: Globin depletion transcript analysis. We represent graphically the proportion of reads (y-axes) correctly classified (green), wrongly targeted by RISER (fuchsia), or for which no decision was made (purple) during the live runs targeting globin mRNA for depletion. We represent these proportions for all globin mRNA, mtRNA and non-globin mRNA (**a**), and for all the individual transcripts from each class: globin mRNA (**b**), mtRNA (**c**) and non-globin mRNA (**d**). Source data are provided as a Source Data file.

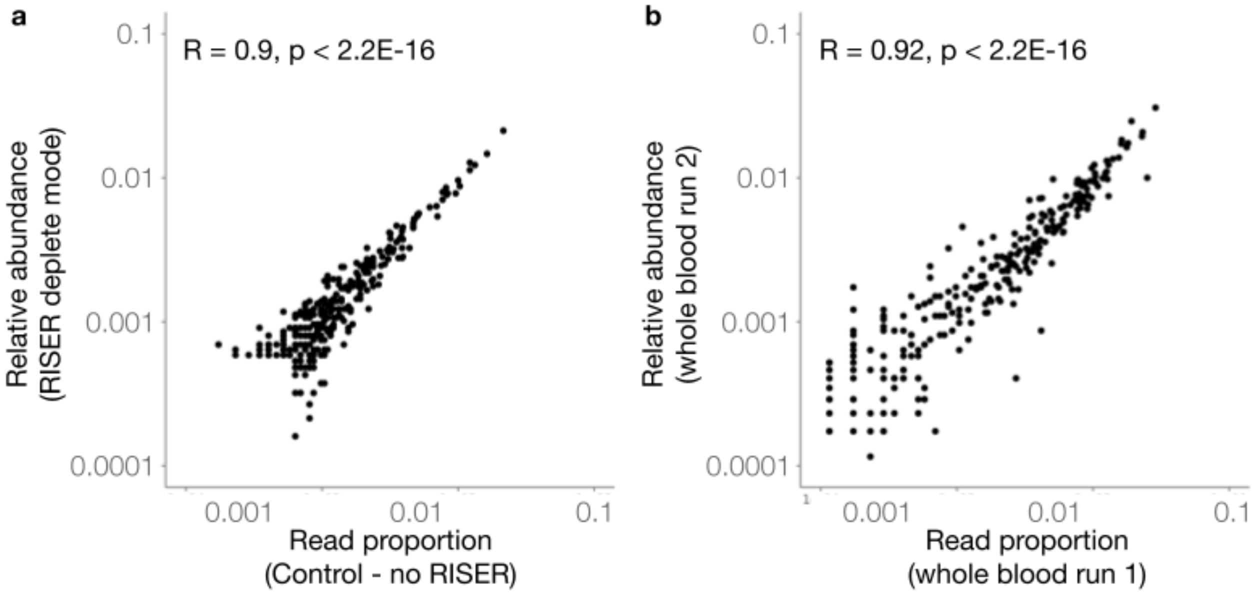

Supplementary Figure 13: Conserved relative abundance of non-globin mRNA after globin depletion. a, For each non-globin mRNA (n = 296) detected in the control (x-axis) and the RISER deplete globin mRNA (y-axis) conditions (in the same flow cell sequencing whole blood), we represent their abundance in log10 scale, measured as the proportion of reads mapped to that non-globin mRNA over all reads mapped to all detected non-globin mRNAs. Only non-globin mRNAs with at least 10 reads in either the control or RISER condition were included. The Pearson correlation *(R)* is also shown. **b,** The abundance (calculated as in (a)) of non-globin mRNAs (n = 285) measured in two separate sequencing experiments of whole blood without RISER, considering the same number of reads as in (a), randomly sampled from the same set of transcripts as in (a). The Pearson correlation *(R)* is also shown. Whole blood samples were prepared and sequenced with a MinION Mk1B as described for the production of globin model training data in Methods. Source data are provided as a Source Data file.

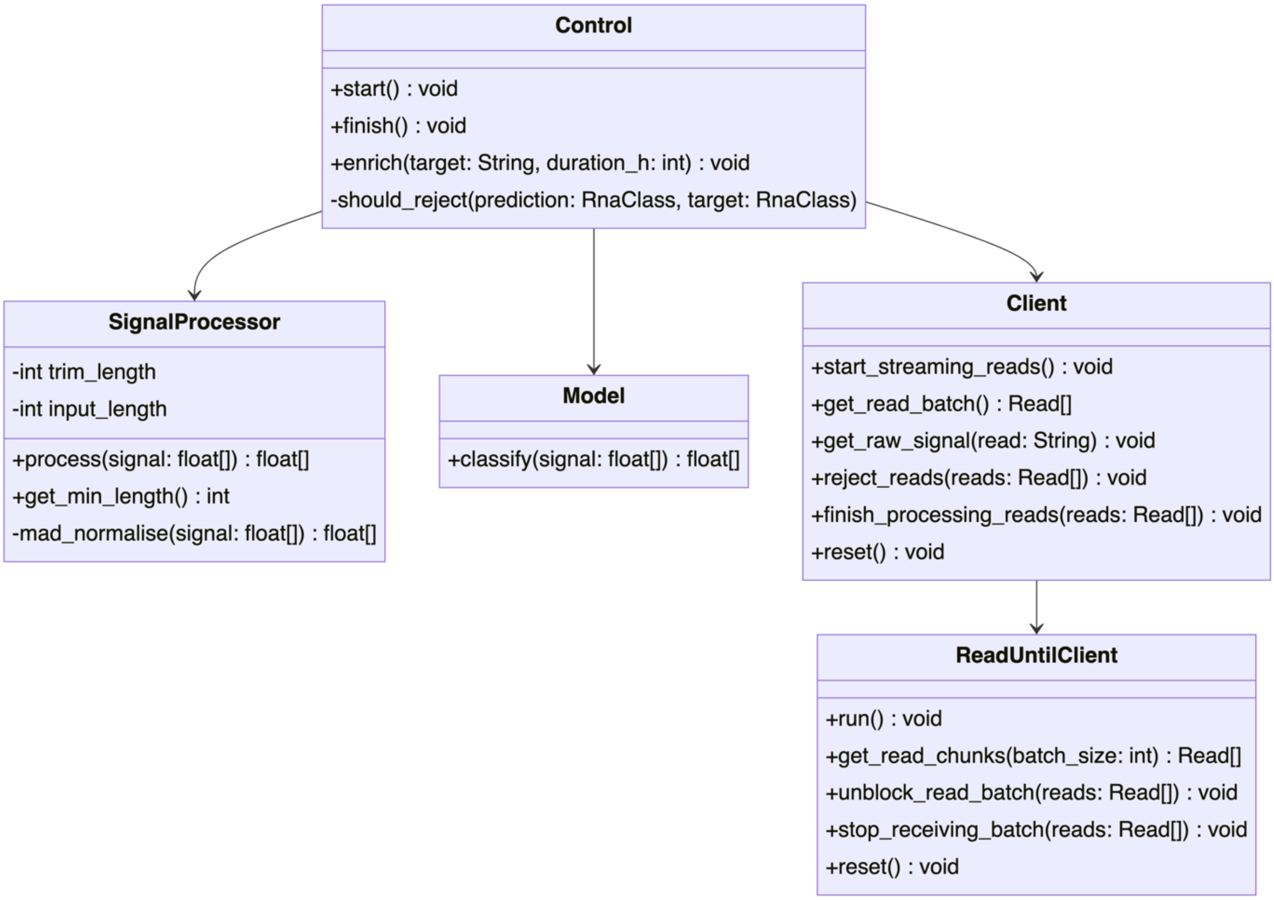

Supplementary Figure 14: Class diagram for the RISER software using unified modelling language (UML) notation. Each box represents a class and contains three sections: name, attributes, and method definitions. Attributes and method definitions have assigned access privileges, with “+” and “-" indicating public and private visibility, respectively. Arrows denote associations between classes, i.e., the class at the arrow's tail has an instance of the class at the tip of the arrow. For simplicity, logging, csv writing functionality and optional method parameters have not been shown. Only methods in the ONT ReadUntilClient (v3.0.0) that have been utilized by RISER are shown.

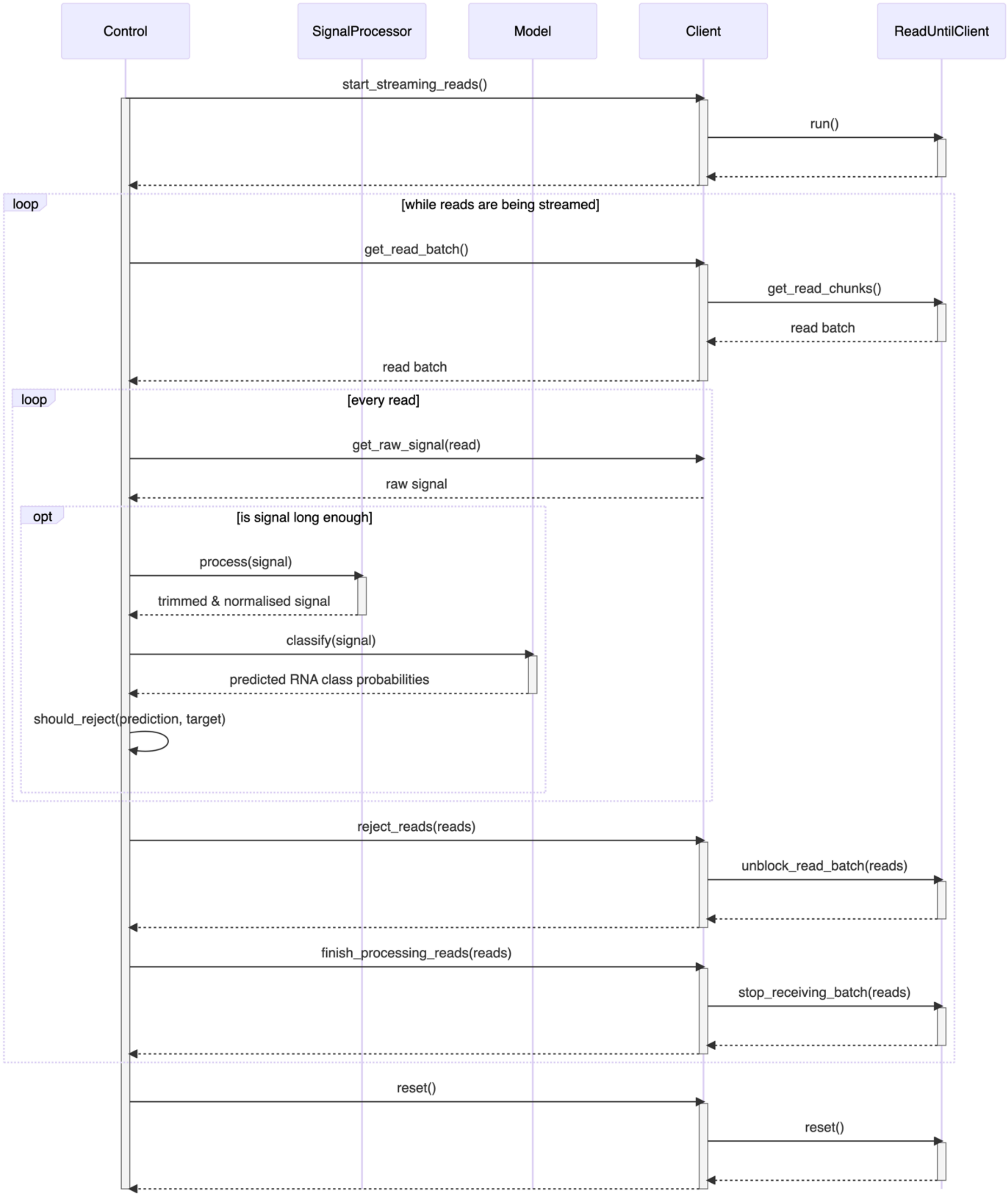

Supplementary Figure 15: Sequence diagram for the RISER software using unified modelling language (UML) notation. The diagram illustrates the timeline (top-to-bottom) of interactions between RISER components during a targeted sequencing run. Each blue box represents an object (class instance), with vertical blue lifelines extending beneath them. The white vertical bars denote when an object is busy (either executing a task or awaiting a message). Arrows denote synchronous messages (the sender requires a reply before continuing), with solid arrows representing messages from the sender to the receiver and dotted arrows denoting the return message from receiver to sender. Conditional functionality is enclosed within an "opt" rectangle, with the condition listed in square brackets. Loops are also enclosed within a "loop" rectangle, with the loop condition listed in square brackets.

Supplementary Tables

Supplementary Table 1: MinION DRS datasets used in this study for the RISER model architecture development. The HEK293-B dataset was composed by taking the second and third biological replicates for both the METTL3 knockout and wild-type samples in ENA PRJEB40872 and combining all technical replicates.

Dataset ID	Cell line / tissue type	Accession #	Sequencing kit	Flow cell	Pore
GM24385	GM24385	TBD	SQK-RNA002 (modified)	FLO-MIN106	R9.4.1
Heart	Human heart	ENA PRJEB40410[2]	SQK-RNA002	FLO-MIN106	R9.4.1
HEK293-A	HEK293	TBD	SQK-RNA002	FLO-MIN106	R9.4.1
HEK293-B	HEK293	ENA PRJEB40872[3]	SQK-RNA002	FLO-MIN106	R9.4.1

Supplementary Table 2: Hyperparameter optimization results for the Residual Network (ResNet) architecture. “O” and “A” denote the “bottleneck” and “basic” residual block types, respectively, defined in [4].

Input signal length	Batch size	Learning rate	Block type	Num. layers (l)	Kernel size (k)	Blocks (b)	Channels (c)	Num. trainable params	Val. accuracy (%)
4s	32	0.001	O	4	13	2,2,2,2	20,30,45,67	75,374	76.44
4s	1000	0.001	O	4	13	2,2,2,2	20,30,45,67	75,374l	75.26
4s	32	0.001	O	4	19	2,2,2,2	16,24,36,54	49,086	76.23
4s	16	0.001	O	4	19	2,2,2,2	20,30,45,67	75,494	76.67
4s	32	0.0001	O	4	19	2,2,2,2	20,30,45,67	75,494	75.41
4s	32	0.001	O	4	19	2,2,2,2	20,30,45,67	75,494	76.59
4s	32	0.001	O	4	19	2,2,2,2	20,30,45,67	24,990	74.68
4s	1000	0.0001	O	4	19	2,2,2,2	20,30,45,67	75,494	73.18
4s	1000	0.001	O	4	19	2,2,2,2	20,30,45,67	75,494	75.41
4s	32	0.001	O	4	19	2,2,2,2	20,40,80,160	278,602	76.8
4s	32	0.001	O	4	19	2,2,2,2	32,48,72,108	191,562	76.75
4s	32	0.001	O	4	19	2,2,2,2	32,64,128,256	708,418	77.08
4s	1000	0.001	O	4	19	2,2,2,2	32,64,128,256	708,418	76.07
4s	32	0.001	O	4	19	2,3,4,2	20,30,45,67	106,154	77.08
4s	1000	0.001	O	4	19	2,3,4,2	20,30,45,67	106,154	75.92
4s	32	0.001	O	4	19	3,4,23,3	20,30,45,67	379,514	78.75
4s	32	0.001	O	4	19	3,4,6,3	128, 256, 512, 1024	7,670,402	77.73
4s	32	0.001	O	4	19	3,4,6,3	20,30,45,67	166,844	78.1
4s	32	0.001	O	4	19	3,4,6,3	256, 512, 1024, 2048	30,596,354	77.43

4s	32	0.001	O	4	19	3,4,6,3	32,64,128,256	487,394	77.94
4s	32	0.001	O	4	19	3,4,6,3	64, 128, 256, 512	1,928,258	78.16
4s	32	0.001	O	4	25	2,2,2,2	20,30,45,67	75,614	76.56
4s	1000	0.001	O	4	25	2,2,2,2	20,30,45,67	75,614	75.35
4s	32	0.001	O	5	19	2,2,2,2,2	20,30,45,67,100	168,384	78.09
4s	1000	0.001	O	5	19	2,2,2,2,2	20,30,45,67,100	168,384	76.8
4s	32	0.001	O	6	19	2,2,2,2,2,2	20,30,45,67,100,150	375,684	79.32
4s	32	0.001	O	7	19	2,2,2,2,2,2,2	20,30,45,67,100,150,225	840,384	80.16
4s	32	0.001	A	4	19	2,2,2,2	20,30,45,67	102,120	78.23
4s	1000	0.001	A	4	19	2,2,2,2	20,30,45,67	102,120	77.14
4s	32	0.001	A	4	19	3,4,6,3	32,64,128,256	2,041,090	79.76
4s	32	0.001	A	4	19	3,4,6,3	64, 128, 256, 512	8,139,266	79.36
4s	32	0.0001	A	10	19	1,1,1,1,1,1,1,1,1,1	20,30,45,67,100,150,225,337,505,757	5,869,558	80.17
4s	**32**	**0.001**	**A**	**10**	**19**	**1,1,1,1,1,1,1,1,1,1**	**20,30,45,67,100,150,225,337,505,757**	**5,869,558**	**81.12**

Supplementary Table 3: Hyperparameter optimization results for the Temporal Convolutional Network (TCN) architecture.

Input signal length	Batch size	Learning Rate	Num. layers (l)	Num. channels (c)	Kernel size (k)	Dilation base (d)	Dropout (r)	# Trainable Params	% Val Accuracy
4s	32	0.0001	5	128	7	6	0.2	1,102,210	77.7
4s	32	0.0001	5	128	7	8	0.2	1,102,210	76.07
4s	32	0.0001	6	128	7	4	0.2	1,348,610	77.54
4s	32	0.0001	7	128	7	3	0.2	1,595,010	76.97

4s	32	0.0001	9	16	15	2	0.2	68,338	76.48
4s	32	0.0001	10	16	7	2	0	37,314	76.35
4s	32	0.0001	10	16	7	2	0.05	37,314	76.56
4s	32	0.0001	10	16	7	2	0.1	37,314	76.29
4s	16	0.0001	10	16	7	2	0.2	37,314	74.89
4s	32	0.00001	10	16	7	2	0.2	37,314	72.46
4s	32	0.0001	10	16	7	2	0.2	37,314	75.17
4s	32	0.001	10	16	7	2	0.2	37,314	72.89
4s	32	0.0001	10	16	9	2	0.2	47,074	76.03
4s	32	0.0001	10	16	11	2	0.2	56,834	76.72
4s	32	0.0001	10	32	7	2	0.2	147,330	76.49
4s	**32**	**0.0001**	**10**	**32**	**11**	**2**	**0.05**	**225,282**	**78.9**
4s	32	0.0001	10	64	7	2	0.2	585,474	77.37
4s	32	0.0001	10	64	11	2	0.05	897,026	78.59
4s	16	0.0001	10	128	7	2	0.2	2,334,210	76.7
4s	32	0.0001	10	128	7	2	0.2	2,334,210	75.76
4s	32	0.0001	11	16	5	2	0.2	30,450	73.68
4s	32	0.0001	12	16	3	2	0.2	21,538	71.23
4s	32	0.0001	12	128	3	2	0.2	1,319,170	75.59

Supplementary Table 4: Hyperparameter optimization results for the "vanilla" Convolutional Neural Network (CNN) architecture. The classifier "fc" denotes a 2-layer fully connected network with rectified linear unit (ReLu) activation, "gap" denotes a global average pooling layer and "gap_fc" denotes a global average pooling layer followed by a single fully connected layer.

Input signal length	Batch size	Learning rate	Num. layers (l)	Blocks per layer (b)	Channels (c)	Kernel size (k)	Classifier (f)	# Trainable params	% Val accuracy
4s	32	0.001	4	1	20,30,45,67	3	fc	206,674,703	69.74
4s	32	0.001	4	1	20,30,45,67	3	gap	15,253	70.08
4s	32	0.001	4	1	20,30,45,67	3	gap_fc	15,523	70.15
4s	32	0.001	4	1	20,30,45,67	5	gap_fc	25,223	72
4s	32	0.001	4	1	20,30,45,67	7	gap_fc	35,193	72.08
4s	32	0.001	4	1	20,30,45,67	11	gap_fc	55,133	73.45
4s	32	0.001	4	1	32,64,128,256	3	gap_fc	130,114	70.79
4s	32	0.001	4	2	20,30,45,67	3	gap_fc	38,857	72.05
4s	32	0.001	4	4	20,30,45,67	3	gap_fc	86,065	50
4s	32	0.0001	4	4	20,30,45,67	3	gap_fc	86,065	73.9
4s	32	0.001	5	1	20,30,45,67,100	3	gap_fc	35,519	72.21
4s	32	0.001	6	1	20,30,45,67,100,150	3	gap_fc	80,769	74.12
4s	32	0.001	7	1	20,30,45,67,100,150,225	3	gap_fc	182,394	50
4s	32	0.0001	7	1	20,30,45,67,100,150,225	3	gap_fc	182,394	76.51
4s	32	0.001	8	1	20,30,45,67,100,150,225,337	3	gap_fc	410,430	50
4s	32	0.0001	8	1	20,30,45,67,100,150,225,337	3	gap_fc	2,069,942	79.38
4s	32	0.0001	8	1	20,40,80,160,320,640,1280,2560	3	gap_fc	13,116,682	78.99
4s	32	0.0001	8	1	32,64,128,256,512,1024,2048,4096	3	gap_fc	33,568,834	79.29

4s	32	0.001	10	1	20,20,30,30,45,45,67,67,100,100	3	gap_fc	89,223	50
4s	32	0.001	10	1	20,30,45,67,100,150,225,337,505,757	3	gap_fc	2,069,942	50
4s	32	0.0001	10	1	20,30,45,67,100,150,225,337,505,757	3	gap_fc	2,069,942	79.35
4s	32	0.0001	10	2	20,30,45,67,100,150,225,337,505,757	3	gap_fc	5,169,924	50
4s	32	0.00001	10	2	20,30,45,67,100,150,225,337,505,757	3	gap_fc	5,169,924	50
4s	32	0.000001	10	2	20,30,45,67,100,150,225,337,505,757	3	gap_fc	5,169,924	50
4s	32	0.0000001	10	2	20,30,45,67,100,150,225,337,505,757	3	gap_fc	5,169,924	50
4s	**32**	**0.0001**	**12**	**1**	**20,30,45,67,100,150,225,337,505,757,1135,1702**	**3**	**gap_fc**	**10,447,564**	**80.22**

Supplementary Table 5: Time taken to classify 1000 fixed-length DRS signals by sequence-based adaptive sampling (AS) and by RISER. AS was performed using basecalling and mapping to the protein-coding transcriptome. Source data are provided as a Source Data file.

Approach	Average classification time per signal (using GPU) (s)	Average classification time per signal (using CPU) (s)
RISER	0.00585	0.0157
AS	0.258	5.83

Supplementary Table 6: MinION DRS datasets used in this study for development and evaluation of the mRNA and mtRNA models. The HEK293-B dataset was composed by taking the second and third biological replicates for both the METTL3 knockout and wild-type samples in ENA PRJEB40872 and combining all technical replicates. Only data from HeLa WT cells was used from GSE211762. (1) All JHU runs 1-5 and (2) Bham run 1 from the Nanopore WGS consortium https://github.com/nanopore-wgs-consortium/NA12878/

Dataset ID	Cell line / tissue type	Accession #	Sequencing kit	Flow cell	Pore	Used for training?
GM12878-A	GM12878	(1)	SQK-RNA001	FLO-MIN106	R9.4.1	Yes
GM12878-B	GM12878	NCBI GEO GSE262285	SQK-RNA002	FLO-MIN106	R9.4.1	Yes
GM12878-C	GM12878	(2)	SQK-RNA001	FLO-MIN106	R9.4.1	No
GM24385	GM24385	NCBI GEO GSE262285	SQK-RNA002 (modified)	FLO-MIN106	R9.4.1	Yes
Heart	Human heart	ENA PRJEB404102	SQK-RNA002	FLO-MIN106	R9.4.1	Yes
HEK293-A	HEK293	NCBI GEO GSE262285	SQK-RNA002	FLO-MIN106	R9.4.1	Yes
HEK293-B	HEK293	ENA PRJEB408724	SQK-RNA002	FLO-MIN106	R9.4.1	Yes
HEK293-C	HEK293	NCBI GEO GSE262285	SQK-RNA002	FLO-MIN106	R9.4.1	Yes
HeLa	HeLa	NCBI GEO GSE262285	SQK-RNA002	FLO-MIN106	R9.4.1	No
KOPN8	KOPN8	NCBI GEO GSE262285	SQK-RNA002	FLO-MIN106	R9.4.1	Yes

REH	REH	NCBI GEO GSE262285	SQK-RNA002	FLO-MIN106	R9.4.1	Yes

Supplementary Table 7: RISER output files - CSV file.

batch_start	read_id	channel	sig_length	prob_targets	threshold	mode	decision
1501	075391a9-2816-45b0-aebb-12b1f398fcd3	204	6878	0.93	0.9	deplete	reject
1501	afcbd456-1843-4322-85d2-7f001ef0dc01	176	7070	0.07	0.9	deplete	accept
1501	d8de6be4-4a01-42dc-b8ab-77abc8f818e1	373	8372	0.11	0.9	deplete	try_again
1502	02cdeae6-3d5d-4615-bc61-7d5dd9a7217c	91	6323	0.89	0.9	deplete	try_again
1502	4460c783-4663-4666-be4d-c52590fdff31	293	10170	0.99	0.9	deplete	reject
	…	…		…	…	…	…

Supplementary Table 8: Wilcoxon signed rank test parameters and statistics. H1 is the alternative hypothesis, U1 is the test statistic and n is the sample size. The probability of superiority (PS) is the effect size, being the probability that a randomly sampled read from the control condition is longer than a randomly sampled read from the deplete condition (i.e., PS close to 0.5 means the lengths are likely to be the same, whereas PS close to 1 means that the control lengths are highly likely to be larger).

Figure	Biotype	Percent change of	H1	*p*	U1	PS	n
Main Fig. 4d	mRNA	Read counts	RISER vs control < between controls	7.9E-287	762,095.0	0.99	1,746
Main Fig. 4d	mtRNA	Read counts	RISER vs control < between controls	3.4E-6	196.0	1.0	28
Main Fig. 4d	lncRNA	Read counts	RISER vs control > between controls	1.6E-4	1.0	0.016	16
Main Fig. 4e	mRNA	Nucleotide counts	RISER vs control < between controls	7.4E-287	762,116.0	0.99	1,746
Main Fig. 4e	mtRNA	Nucleotide counts	RISER vs control < between controls	3.4E-6	196.0	1.0	28
Main Fig. 4e	lncRNA	Nucleotide counts	RISER vs control > between controls	5.4E-4	3.0	0.046	16
Main Fig. 5f	Globin mRNA	Read counts	RISER vs control < between controls	0.014	16.0	1.0	8
Main Fig. 5f	Non-globin mRNA	Read counts	RISER vs control > between controls	3.6E-6	3,164.5	0.32	200
Main Fig. 5g	Globin mRNA	Nucleotide counts	RISER vs control < between controls	0.014	16.0	1.0	8
Main Fig. 5g	Non-globin mRNA	Nucleotide counts	RISER vs control > between controls	3.6E-5	3,375.0	0.34	200

Supp. Fig. 7a	mRNA	Read counts	RISER vs control < between controls	2.1E-262	636,792.5	0.99	1,596
Supp. Fig. 7a	mtRNA	Read counts	RISER vs control < between controls	3.4E-6	196.0	1.0	28
Supp. Fig. 7a	lncRNA	Read counts	RISER vs control > between controls	2.2E-3	1.0	0.028	12
Supp. Fig. 7b	mRNA	Nucleotide counts	RISER vs control < between controls	2.0E-262	636,797.0	0.99	1,596
Supp. Fig. 7b	mtRNA	Nucleotide counts	RISER vs control < between controls	3.4E-6	196.0	1.0	28
Supp. Fig. 7b	lncRNA	Nucleotide counts	RISER vs control > between controls	0.013	4.0	0.013	12
Supp. Fig. 11a	Globin mRNA	Read counts	RISER vs control < between controls	0.014	16.0	1.0	8
Supp. Fig. 11a	Non-globin mRNA	Read counts	RISER vs control > between controls	2.3E-6	2,643.0	0.31	186
Supp. Fig. 11b	Globin mRNA	Nucleotide counts	RISER vs control < between controls	0.014	16.0	1.0	8
Supp. Fig. 11b	Non-globin mRNA	Nucleotide counts	RISER vs control > between controls	2.9E-6	2,660.0	0.31	186

Supplementary Notes

Supplementary Note 1: RISER command structure.

```
usage: riser.py [-h] -t -m -d [--min] [--max] [--threshold]


optional arguments:
 -h, --help          show this help message and exit
 -t, --target        RNA class to enrich for. This must be one or more of
                     {mRNA,mtRNA,globin}. (required)
 -m, --mode          Whether to enrich or deplete the target class. This
                     must be one of {enrich,deplete} (required)
 -d, --duration      Length of time (in hours) to run RISER for. This
                     should be the same as the MinKNOW run length.
                     (required)
 --min               Minimum number of seconds of transcript signal to use
                     for RISER prediction. (default: 2)
 --max               Maximum number of seconds of transcript signal to try
                     to classify before skipping this read. (default: 4)
 --threshold         Probability threshold for classifier [0,1]. (default:
                     0.9)
```

Supplementary Note 2: RISER output files - Console output.

```
Using cuda device
Usage: riser.py --target mRNA mtRNA --mode deplete --duration 24
All settings used (including those set by default):
--target        : ['mRNA', 'mtRNA']
--mode          : deplete
--duration_h    : 24
--min           : 2
--max           : 4
--threshold     : 0.9
Client is running.
Live read stream started.
In the last minute 774 signals were assessed, 21 were accepted and 636
were rejected
In the last minute 767 signals were assessed, 36 were accepted and 621
were rejected
In the last minute 801 signals were assessed, 28 were accepted and 637
were rejected
In the last minute 811 signals were assessed, 34 were accepted and 671
were rejected
...
```

Supplementary Note 3: RISER output files - Log file.

```
2023-12-06T16:59:16 [minknow_api] DEBUG: Reading ssl certificate
2023-12-06T16:59:16 [minknow_api] DEBUG: Getting local token
2023-12-06T16:59:17 [minknow_api] DEBUG: Retrieving local token from file:
'/tmp/minknow-auth-token.json'
2023-12-06T16:59:17 [minknow_api] DEBUG: Calling get_version_info to test
connection
2023-12-06T16:59:17 [RISER] INFO: Using cuda device
2023-12-06T16:59:20 [RISER] INFO: Using cuda device
```

```
2023-12-06T16:59:20 [RISER] INFO: Usage: riser.py --target mRNA mtRNA --
mode deplete --duration 24
2023-12-06T16:59:20 [RISER] INFO: All settings used (including those set
by default):
2023-12-06T16:59:20 [RISER] INFO: --target        : ['mRNA', 'mtRNA']
2023-12-06T16:59:20 [RISER] INFO: --mode          : deplete
2023-12-06T16:59:20 [RISER] INFO: --duration_h    : 24
2023-12-06T16:59:20 [RISER] INFO: --min           : 2
2023-12-06T16:59:20 [RISER] INFO: --max           : 4
2023-12-06T16:59:20 [RISER] INFO: --threshold     : 0.9
2023-12-06T16:59:20 [RISER] INFO: Client is running.
2023-12-06T16:59:20 [RISER] INFO: Live read stream started.
2023-12-06T17:00:20 [RISER] INFO: In the last minute 774 signals were
assessed, 21 were accepted and 636 were rejected
2023-12-06T17:01:20 [RISER] INFO: In the last minute 767 signals were
assessed, 36 were accepted and 621 were rejected
2023-12-06T17:02:20 [RISER] INFO: In the last minute 801 signals were
assessed, 28 were accepted and 637 were rejected
2023-12-06T17:03:20 [RISER] INFO: In the last minute 811 signals were
assessed, 34 were accepted and 671 were rejected
...
```

Supplementary references

1. Sneddon, A., Mateos, P. A., Shirokikh, N. E. & Eyras, E. Language-Informed Basecalling Architecture for Nanopore Direct RNA Sequencing. *Proceedings of the 17th Machine Learning in Computational Biology meeting* **PMLR 200**, 150–165 (2022).

2. de la Rubia, I. *et al.* RATTLE: reference-free reconstruction and quantification of transcriptomes from Nanopore sequencing. *Genome Biol* **23**, 153 (2022).

3. Pratanwanich, P. N. *et al.* Identification of differential RNA modifications from nanopore direct RNA sequencing with xPore. *Nat Biotechnol* **39**, 1394–1402 (2021).

4. He, K., Zhang, X., Ren, S. & Sun, J. Deep Residual Learning for Image Recognition. in *2016 IEEE Conference on Computer Vision and Pattern Recognition (CVPR)* 770–778 (IEEE, 2016). doi:10.1109/CVPR.2016.90.
