# Supplementary material for: Biochemical-free enrichment or depletion of RNA classes in real-time during direct RNA sequencing with RISER
Source: Nat Commun. 2024 May 24;15:4422. doi: 10.1038/s41467-024-48673-8 (PMC11126589; doi:10.1038/s41467-024-48673-8)
Supplement: Supplementary file 3 — Reporting Summary [file 41467_2024_48673_MOESM3_ESM.pdf]

nature portfolio

Corresponding author(s): Nikolay E. Shirokikh, Eduardo Eyras

Last updated by author(s): Mar 20, 2024

# Reporting Summary

Nature Portfolio wishes to improve the reproducibility of the work that we publish. This form provides structure for consistency and transparency in reporting. For further information on Nature Portfolio policies, see our Editorial Policies and the Editorial Policy Checklist.

## Statistics

For all statistical analyses, confirm that the following items are present in the figure legend, table legend, main text, or Methods section.

| n/a | Confirmed | |
|---|---|---|
| ☐ | ☒ | The exact sample size (*n*) for each experimental group/condition, given as a discrete number and unit of measurement |
| ☐ | ☒ | A statement on whether measurements were taken from distinct samples or whether the same sample was measured repeatedly |
| ☐ | ☒ | The statistical test(s) used AND whether they are one- or two-sided<br>*Only common tests should be described solely by name; describe more complex techniques in the Methods section.* |
| ☒ | ☐ | A description of all covariates tested |
| ☐ | ☒ | A description of any assumptions or corrections, such as tests of normality and adjustment for multiple comparisons |
| ☐ | ☒ | A full description of the statistical parameters including central tendency (e.g. means) or other basic estimates (e.g. regression coefficient) AND variation (e.g. standard deviation) or associated estimates of uncertainty (e.g. confidence intervals) |
| ☐ | ☒ | For null hypothesis testing, the test statistic (e.g. *F*, *t*, *r*) with confidence intervals, effect sizes, degrees of freedom and *P* value noted<br>*Give P values as exact values whenever suitable.* |
| ☒ | ☐ | For Bayesian analysis, information on the choice of priors and Markov chain Monte Carlo settings |
| ☒ | ☐ | For hierarchical and complex designs, identification of the appropriate level for tests and full reporting of outcomes |
| ☐ | ☒ | Estimates of effect sizes (e.g. Cohen's *d*, Pearson's *r*), indicating how they were calculated |

*Our web collection on statistics for biologists contains articles on many of the points above.*

## Software and code

Policy information about availability of computer code

| | |
|---|---|
| Data collection | wget (freely available from the GNU project under the GPL-v3 license), SRA toolkit (freely available from https://github.com/ncbi/sra-tools under a public domain license) |
| Data analysis | RISER (commit 30d7062): https://github.com/comprna/riser/tree/30d70622b0bb1415a96daff87fed045567e36672<br>Minimap2 (v2.1.0): https://github.com/lh3/minimap2<br>Guppy (v4.0.14): Commercial software from Oxford Nanopore Technologies<br>Samtools (v1.10): https://github.com/samtools/samtools<br>PyTorch (v1.9.0): https://github.com/pytorch/pytorch<br>BoostNano (commit 8715800): https://github.com/haotianteng/BoostNano<br>MinKNOW (standalone GUI v5.7.10, core v5.7.2): Commercial software from Oxford Nanopore Technologies<br>ReadUntil API (v3.4.1): https://github.com/nanoporetech/read_until_api<br>MinKNOW API (v5.5.2): https://github.com/nanoporetech/minknow_api |

For manuscripts utilizing custom algorithms or software that are central to the research but not yet described in published literature, software must be made available to editors and reviewers. We strongly encourage code deposition in a community repository (e.g. GitHub). See the Nature Portfolio guidelines for submitting code & software for further information.

## Data

Policy information about availability of data

All manuscripts must include a data availability statement. This statement should provide the following information, where applicable:

- Accession codes, unique identifiers, or web links for publicly available datasets
- A description of any restrictions on data availability
- For clinical datasets or third party data, please ensure that the statement adheres to our policy

All datasets used in this study are publicly available. The nanopore DRS signals generated in this study (GM12878-B, GM24385, HEK293-A, HEK293-C, HeLa, KOPN8, REH and whole blood) have been deposited in the NCBI Gene Expression Omnibus (GEO) database under accession code GSE262285 [https://www.ncbi.nlm.nih.gov/geo/query/acc.cgi?acc=GSE262285]. The nanopore DRS signals for GM12878 cells used in this study are available in the Nanopore Whole Genome Sequencing Consortium (https://github.com/nanopore-wgs-consortium/NA12878/) Johns Hopkins University (all runs) (GM12878-A) and University of Birmingham (run 1) (GM12878-C). The nanopore DRS signals for human heart used in this study are available in the European Nucleotide Archive (ENA) under accession code PRJEB40410. The nanopore DRS signals for HEK293 cells (HEK293-B) used in this study are available in the ENA under accession code PRJEB40872. Source data are provided with this paper.

## Research involving human participants, their data, or biological material

Policy information about studies with human participants or human data. See also policy information about sex, gender (identity/presentation), and sexual orientation and race, ethnicity and racism.

| | |
|---|---|
| Reporting on sex and gender | Sex and gender information has not been collected and is not relevant to this study. |
| Reporting on race, ethnicity, or other socially relevant groupings | Race, ethnicity, nor any other social groupings have been collected and are not relevant to this study. |
| Population characteristics | Population characteristics were not collected and were not relevant to this study. |
| Recruitment | N/A |
| Ethics oversight | Blood samples were obtained from three human donors with informed consent and approval from the ethics committee from the Australian National University (ANU) under ethics protocol no. ETH.1.16.01/ETH.01.15.015 |

Note that full information on the approval of the study protocol must also be provided in the manuscript.

# Field-specific reporting

Please select the one below that is the best fit for your research. If you are not sure, read the appropriate sections before making your selection.

[x] Life sciences   [ ] Behavioural & social sciences   [ ] Ecological, evolutionary & environmental sciences

For a reference copy of the document with all sections, see nature.com/documents/nr-reporting-summary-flat.pdf

# Life sciences study design

All studies must disclose on these points even when the disclosure is negative.

| | |
|---|---|
| Sample size | To train and test the RISER models during development, the sample size of our training dataset was selected to maximise the number of signals and diversity of transcripts. We used all the direct RNA sequencing runs from human tissues or cell lines that were available to us for training. The final sample size of the training and testing sets were determined after balancing the datasets equally between classes, also preserving a balance of biotypes within each class, as described in the Methods section.<br><br>To test the RISER models in real-time during live MinION sequencing, we performed sequencing over 24 hours, to ensure enough reads were acquired and assessed by RISER, so that a sufficient sample size could be used to evaluate RISER's impact, as described in the Methods section. |
| Data exclusions | For training, we excluded the following RNA biotypes: Pseudogenes, artifact, non_stop_decay, nonsense_mediated_decay, processed_transcript, retained_intron and TEC. These biotypes were excluded to ensure there were no common sequences between the two classes, as indicated in the Methods section.<br><br>For lncRNA analysis, we excluded lncRNAs that overlap mRNAs in protein-coding genes, as described in the Methods section. |
| Replication | We tested RISER once on HeLa cells (produced by us) and once on sequencing data from GM12878 cells produced by a different lab (based on a different sequencing kit), as described in the Methods section.<br><br>Testing RISER in real-time during live sequencing was performed once using HEK293 cells (produced by us) and once using a whole blood sample (collected by us), with the performance in each was compared against two control run (no RISER) replicates for HEK293 cells and whole blood, respectively, as described in the Methods. section. |

| | |
|---|---|
| | The variability in pore degradation over time was evaluated across 6 different sequencing runs, as described in the Methods section. |
| Randomization | Randomization was performed at the time of splitting the datasets into training, validation, and testing, as described in the Methods section. |
| Blinding | Blinding was not performed in this study, since the sequencing was performed on samples of known origin. |

# Reporting for specific materials, systems and methods

We require information from authors about some types of materials, experimental systems and methods used in many studies. Here, indicate whether each material, system or method listed is relevant to your study. If you are not sure if a list item applies to your research, read the appropriate section before selecting a response.

## Materials & experimental systems

| n/a | Involved in the study | |
|---|---|---|
| ☒ | ☐ | Antibodies |
| ☐ | ☒ | Eukaryotic cell lines |
| ☒ | ☐ | Palaeontology and archaeology |
| ☒ | ☐ | Animals and other organisms |
| ☒ | ☐ | Clinical data |
| ☒ | ☐ | Dual use research of concern |
| ☒ | ☐ | Plants |

## Methods

| n/a | Involved in the study | |
|---|---|---|
| ☒ | ☐ | ChIP-seq |
| ☒ | ☐ | Flow cytometry |
| ☒ | ☐ | MRI-based neuroimaging |

## Eukaryotic cell lines

Policy information about cell lines and Sex and Gender in Research

| | |
|---|---|
| Cell line source(s) | HeLa cells (human cervical cancer) were obtained from ATCC (cat. no. CCL-2)<br>GM12878 cells (immortalized human peripheral vein-derived B-cells) were obtained from the Coriell Institute<br>GM24385 cells (lymphoblastoid cell line) were obtained from the Coriell Institute<br>HEK293 cells (human embryonic kidney) were obtained from ATCC (cat. no. CRL-1573)<br>KOPN8 cells (B-cell acute lymphocytic leukemia) were obtained from DSMZ (cat. no. ACC 552)<br>REH cells (B-cell acute lymphocytic leukemia) were obtained from DSMC (cat. no. ACC 22) |
| Authentication | Cell lines were confirmed via short tandem repeat (STR) profiling with CellBank Australia. |
| Mycoplasma contamination | All cell lines were tested by the commercial provider and were negative for mycoplasma contamination prior to being shipped. Further, all cell lines were used in this study shortly after receipt, with minimal passages undertaken. |
| Commonly misidentified lines (See ICLAC register) | No commonly misidentified cell lines were used in this study. |

## Plants

| | |
|---|---|
| Seed stocks | *Report on the source of all seed stocks or other plant material used. If applicable, state the seed stock centre and catalogue number. If plant specimens were collected from the field, describe the collection location, date and sampling procedures.* |
| Novel plant genotypes | *Describe the methods by which all novel plant genotypes were produced. This includes those generated by transgenic approaches, gene editing, chemical/radiation-based mutagenesis and hybridization. For transgenic lines, describe the transformation method, the number of independent lines analyzed and the generation upon which experiments were performed. For gene-edited lines, describe the editor used, the endogenous sequence targeted for editing, the targeting guide RNA sequence (if applicable) and how the editor was applied.* |
| Authentication | *Describe any authentication procedures for each seed stock used or novel genotype generated. Describe any experiments used to assess the effect of a mutation and, where applicable, how potential secondary effects (e.g. second site T-DNA insertions, mosiacism, off-target gene editing) were examined.* |
